# Annotations

**Published:** 1903-12-26

**Authors:** 


### Annotations.

### Fashion in Foods.

Now that our hoardings are placarded with enormous pictorial advertisements of the merits of various kinds of cereal food, someone has risen to denounce the first and best known of all—oatmeal. The complainant declares that the prevalence of rickets in Glasgow is due to the consumption of oatmeal porridge. Such a statement is rather beyond what the facts warrant. If he had said that rickets was more common in Scotland than elsewhere there might be some ground for the arraignment of oatmeal, but ignoring the Highland glens and isolated farms where the people are, as the late Robert Wallace, M.P., said he was in his childhood, "compulsory vegetarians," and yet grow to strong and vigorous maturity of mind and body, he chooses an industrial town where the conditions of life cannot be so healthy, and where even the poorest are more likely to indulge in a varied diet. As a matter of fact the prevalence of rickets in Glasgow has been set down by local observers to the growing luxury of the working classes, which has led to a diminution in the consumption of porridge. Children whose parents had porridge for breakfast, and often for supper also, now subsist on tea and white bread, with scraps of meat, often of a kind more savoury than nourishing. There is no proportionate increase in the consumption of oatmeal to account for an increase in the prevalence of rickets. It does not follow, however, that oatmeal, whatever its merits, is invariably the best food for breakfast or any other meal. Moreover, its consumption tends to a temporary sense of repletion, which prevents the eating of any further food, while hunger often comes on before the time of the next meal. In short, it is not the best food for everybody, and perhaps because it has been so thrust upon people by its advocates, it is doomed to the inevitable reaction, and will for a little while be unjustly decried.

### An Apology for Cheapness.

A brilliant woman writer has lately declared that our humbler classes are demoralised by the cheapness of dress in this country, being thereby tempted to indulge in an endless amount of trumpery adornment, and concludes that if, by increasing the cost of dress, we can raise its standard, we may thereby elevate the national character. The idea is an attractive one, and at the present time is likely to be popular. Cotton-backed satin and shoddy stuffs do not appeal to people of taste. But cheapness means the possibility of change, and change in clothing is desirable from other reasons than mere vanity. In the Middle Ages even the respectable wore the same undergarments by night and by day, and the rich bequeathed to their heirs half-worn robes which, from the nature of the fabric, could not be cleansed by any method then known. That many of these proved as fatal to the recipients as the robe of Deianeira to Hercules, and held infection in their folds there is much reason to believe. It is possible that our poorest still cling too long to garments which have some pretence to respectability, and if the artisan's wife, besides having sufficient change of underclothing for her family, also desires for them new outer garments in order to be "in the fashion" she is, albeit unconsciously, acting in the interests of health and cleanliness. There is, even now, more cause to complain of the outer clothing of all classes being worn too long for perfect sanitation than of the changes which cheapness makes possible. The economic preference which prevails among us for dark clothes which do not show the dirt is hardly compatible with ideal cleanliness. There would be, at least on some points, cause for regret if the price of clothes were to be raised to such a point as to make it less easy for the poorer classes to get a reasonably frequent change of attire. Moreover—though this is indeed in every way a thing to be regretted—the best things do not always provide the best reward for those who make them. The shoddy gown or suit, which is one of hundreds alike in every respect, is made in a factory where the conditions of labour are at least reasonably good, and where the work itself is often more regular than it is with more fashionable firms It is in the plainest work that the fluctuations of trade, its seasons of over-pressure alternating with slackness, are least felt.

### Women and Betting.

A writer in a contemporary narrates how a Lancashire working woman told him that one reason why trade was bad in her neighbourhood was that "the women bet a shilling on nearly every race." This is not, strictly speaking, a reason for bad trade, but it is a reason for that distress which usually accompanies bad trade, and it was doubtless in this sense that the woman meant it. The habit of betting among women of the lower class is a comparatively new vice, but it is one which has grown with amazing rapidity, especially in the towns and villages of our industrial districts. Moreover, no power of law seems to avail much against the vice. It is doubtful if drunkenness is increasing among our working people, but there seems little doubt that betting is. That either or both of these vices should be spreading among women is a serious feature of national life. It is fashionable to sneer at the idea of having one standard of virtue for men and another for women, but if we still hold the English idea that the home is the nursery of civic virtue, it must be admitted that the vices of women have a more serious effect on national character than those of men, because they so much more deeply injure the home. The mother who bets or drinks—and often enough the two faults go together—not only robs her children of their bread, but of something higher, of those ideals of purity, industry, and self-control, which should inspire them in turn throughout their life. Good men and noble women may be reared in a home where the father's faults give the more room for the mother's virtues, but very rarely do they come from one that has been degraded or destroyed by the vices of a mother. Undoubtedly the cause of the development of both drinking and betting among women of the working class is the amount of wealth which our national prosperity has scattered among people whose ideas, even of pleasure, are not of a very high order. One would fain see a better standard of life accompany this prosperity and turn it to higher uses, but if this may not be, the thoughtful may find cause for consolation even in a temporary diminution of national wealth.

